# Milk phospholipids protect *Bifidobacterium longum* subsp. *infantis* during *in vitro* digestion and enhance polysaccharide production

**DOI:** 10.3389/fnut.2023.1194945

**Published:** 2023-11-06

**Authors:** Erica Kosmerl, Brianda D. González-Orozco, Israel García-Cano, Joana Ortega-Anaya, Rafael Jiménez-Flores

**Affiliations:** ^1^Department of Food Science and Technology, The Ohio State University, Columbus, OH, United States; ^2^Department of Food Science and Technology, National Institute of Medical Sciences and Nutrition Salvador Zubirán, Mexico City, Mexico; ^3^Arla Innovation Centre, Aarhus, Denmark

**Keywords:** milk phospholipids, bound polysaccharides, probiotic, bifidobacteria, *in vitro* digestion, adhesion

## Abstract

*Bifidobacterium longum* subsp. *infantis* is associated with the gut microbiota of breast-fed infants. *Bifidobacterium infantis* promotes intestinal barrier and immune function through several proposed mechanisms, including interactions between their surface polysaccharides, the host, and other gut microorganisms. Dairy foods and ingredients are some of the most conspicuous food-based niches for this species and may provide benefits for their delivery and efficacy in the gut. Milk phospholipid (MPL)-rich ingredients have been increasingly recognized for their versatile benefits to health, including interactions with the gut microbiota and intestinal cells. Therefore, our objective was to investigate the capacity for MPL to promote survival of *B. infantis* during simulated digestion and to modulate bacterial polysaccharide production. To achieve these aims, *B. infantis* was incubated with or without 0.5% MPL in de Man, Rogosa, and Sharpe (MRS) media at 37°C under anaerobiosis. Survival across the oral, gastric, and intestinal phases using *in vitro* digestion was measured using plate count, along with adhesion to goblet-like intestinal cells. MPL increased *B. infantis* survival at the end of the intestinal phase by at least 7% and decreased adhesion to intestinal cells. The bacterial surface characteristics, which may contribute to these effects, were assessed by ζ-potential, changes in surface proteins using comparative proteomics, and production of bound polysaccharides. MPL decreased the surface charge of the bifidobacteria from −17 to −24 mV and increased a 50 kDa protein (3-fold) that appears to be involved in protection from stress. The production of bound polysaccharides was measured using FTIR, HPLC, and TEM imaging. These techniques all suggest an increase in bound polysaccharide production at least 1.7-fold in the presence of MPL. Our results show that MPL treatment increases *B. infantis* survival during simulated digestion, induces a stress resistance surface protein, and yields greater bound polysaccharide production, suggesting its use as a functional ingredient to enhance probiotic and postbiotic effects.

## 1. Introduction

Orally-delivered probiotics and postbiotics are some of the most widely accepted and used therapeutics to establish, restore, and maintain gut health (1). Probiotics are defined as “live microorganisms that, when administered in adequate amounts, confer a health benefit on the host” (2). On the other hand, postbiotics are defined as “inanimate microorganisms and/or their components that confer a health benefit on the host” (3). Bifidobacteria are a group of gram-positive, non-motile, anaerobic bacteria and are associated with the consumption of human breast milk in infants due to their ability to catabolize complex carbohydrates (i.e., host mucin glycans, human milk oligosaccharides) through the fermentation pathways known as the “bifid shunt.” The populations of bifidobacteria in the gut microbiota decline with age and the introduction of new foods. In general, a high abundance of these bacteria in the gut is associated with a healthy gut microbiota, whereas low abundances are linked to cystic fibrosis, hepatitis B, cardiometabolic diseases, and more (4).

The benefit of bifidobacteria in the gastrointestinal tract (GIT) is attributed to their metabolic capabilities as well as their stimulatory roles at the host’s mucosal barrier. Bifidobacteria possess the metabolic machinery to synthesize conjugated linoleic acids, polyphenols, B and K vitamins, lantibiotics, and polysaccharides. Bifidobacterial polysaccharides can be classified into the following two types: (i) cell wall polysaccharides (CWP), which are anchored to the bacterial cell wall and attached to the peptidoglycan; and (ii) extracellular polysaccharides (EPS) that can be either released to the medium or form a capsule around the cell (capsular EPS). In this study, bound polysaccharides can be defined as those that are associated with the bacterial cell surface. *Bifidobacterium* species predominantly produce heteropolysaccharides composed of D-glucose, D-galactose, and D-rhamnose, and a molecular weight between 10^4^ and 10^6^ Da; however, the ratio and composition of sugars varies between strains (5). Bifidobacteria polysaccharides have shown postbiotic potential through their immunomodulatory, antitumoral, antimicrobial, and antioxidant activities (6). Recently polysaccharides have been described as one of the most potent modulators of the gut microbiota composition (7). As such, their role in regulating the human gut microbiota by the production of propionic acid was also reported (8). In addition to these functions of bifidobacterial polysaccharides as postbiotics, these polysaccharides also aid in probiotic potential of bifidobacteria through increased tolerance to the GIT conditions (9).

Current trends to prolong bifidobacteria viability as probiotics include encapsulation (i.e., whey proteins, alginates), addition of bioactive components, and incorporation in dairy food matrices (10–12). The milk fat globule membrane (MFGM) is one bioactive component in mammalian milk that emulsifies fat globules with a unique trilayer structure, and has a positive impact on the abundance of bifidobacteria in the intestines of infants and murine models (4, 13). The MFGM is composed of primarily 5 major classes of phospholipids and hundreds of different glycoproteins (14). Its phospholipid constituents, generally referred to as milk phospholipids or MPL, include phosphatidylcholine (PC), phosphatidylethanolamine (PE), sphingomyelin (SM), phosphatidylinositol (PI), and phosphatidylserine (PS). The MFGM confers numerous benefits to health and narrows the developmental gap between formula-fed and breast-fed infants. Specifically, it boosts brain development and neurocognitive function (15, 16), as well as enhances gut barrier function (17) in double-blinded clinical trials. Furthermore, evidence suggests that the MFGM is capable of preventing cardiometabolic diseases (18), modulating bone remodeling (19), and inhibiting enteric pathogens (i.e., *Escherichia coli*, *Salmonella* spp., *Listeria monocytogenes*) (20–22). Among this plethora of health benefits, MFGM may also enrich the abundance of *Bifidobacterium* (4, 13) and prolong their longevity in the GIT, as well as improve their probiotic efficacy.

Based on this evidence, we hypothesized that MPL could modify the survival and adhesion of *Bifidobacterium longum* subsp. *infantis* by increasing polysaccharide production. The aims of this work were: (i) to assess the impact of MPL on survival of *B. infantis* throughout *in vitro* digestion using a consensus model (23) and to quantify changes in adhesion to human intestinal goblet-like cells; and (ii) to determine the effect of MPL on bacterial surface properties, including changes in surface charge, bound polysaccharides, and surface proteins.

## 2. Materials and methods

### 2.1. Milk phospholipids and bacterial media preparation

An MPL ingredient originally isolated from MFGM beta-serum powder was generously donated from Fonterra Co-operative Group Ltd. (PL700 Concentrate, Auckland, NZ). The major phospholipid classes and fatty acid composition of this ingredient are described in Supplementary Table S1. For bacterial growth, de Man Rogosa Sharpe (MRS) broth (Sigma Aldrich, St. Louis, MO) supplemented with 0.05% L-cysteine (Thermo Fisher, Waltham, MA; referred to as MRSC) was prepared with or without 0.5% MPL added prior to autoclaving based on preliminary experiments, which determined 0.5% was suitable for bacterial growth. MRSC agar plates were also prepared using 1.5% agar.

### 2.2. Bacterial growth conditions

*Bifidobacterium longum* subsp. *infantis* ATCC 15697 (*B. infantis*) was purchased from the American Type Culture Collection (ATCC, Manassas, VA). The strain was inoculated from glycerol stocks stored at −80°C to MRSC broth and covered with sterile mineral oil (Bio-Rad, Hercules, CA) for anaerobiosis at 37°C. Following two subcultures, the bacteria was adjusted to an optical density (OD_600_) of 0.1 ± 0.01 corresponding to ~10^8^–10^9^ CFU/mL on a microplate reader (Multiskan GO, Thermo Fisher) and added at 10% of the final volume to MRSC with or without MPL (0.5% w/v). *Bifidobacterium infantis* was incubated with or without MPL in anaerobic conditions at 37°C for approximately 15 h to reach the late exponential growth phase. After treatment, each strain was washed 3× using sterile saline solution (0.85% NaCl (w/v), pH 7.0) and the suspension was again adjusted to OD_600_ = 0.1 ± 0.01.

### 2.3. Survival during *in vitro* digestion

The effect of MPL on the survival of *B. infantis* during simulated digestion was tested following the static INFOGEST 2.0 digestion model and guidelines consisting of oral, gastric, and intestinal phases (23). For the oral phase, 5 mL of bacterial suspension (OD_600_ = 0.1) was mixed with 4 mL of simulated salivary fluid, 1.5 mM CaCl_2_, 75 U/mL salivary α-amylase, and distilled water up to a final volume of 10 mL. The oral phase was incubated at 37°C for 2 min with mixing on a rocking platform (VWR, Radnor, PA) at 95 rpm. For the gastric phase, 8 mL of simulated gastric fluid was added to the same tube and pH was adjusted to 3.0 using 5 M HCl. Then, 2,000 U/mL pepsin, 0.15 mM CaCl_2_, and distilled water up to final volume of 20 mL were added and tubes were incubated at 37°C for 2 h with mixing. For the intestinal phase, 8 mL of simulated intestinal fluid was added to each tube after the oral and gastric phases and pH was adjusted to 7.0 using 5 M NaOH. Tubes were incubated with porcine bile salts (10 mM) for 30 min with rocking. Following bile salt incubation, pancreatin (trypsin activity 100 U/mL), 0.6 mM CaCl_2_, and water up to a final volume of 40 mL were added and incubated for 2 h at 37°C. Preliminary experiments were conducted to determine the appropriate volumes of 5 M HCl and 5 M NaOH for pH adjustments. Throughout the simulated digestion, partially anaerobic conditions were maintained using a flush of N_2_ gas prior to incubation. Individual tubes were prepared for each timepoint to reduce the effects of repeated sampling. At the end of incubation, the digesta was immediately centrifuged (3,500 × g, 4°C, 10 min), followed by removal of the supernatant, resuspension of the pellet in 5 mL of saline solution, and plating on MRSC agar plates using serial dilutions. All digestion reagents were purchased from Sigma.

### 2.4. HT29-MTX cell culture

The ability of MPL-treated *B. infantis* to adhere to human goblet-like cells was investigated using the HT29-MTX-E12 goblet-like cell line (European Collection of Authenticated Cell Cultures, Salisbury, United Kingdom). HT29-MTX cells were cultured in 25 mM high glucose Dulbecco’s Modified Eagle Medium (DMEM) containing 10% heat-inactivated fetal bovine serum, 1% penicillin–streptomycin (100 units/mL penicillin and 100 units/mL streptomycin), 1% non-essential amino acids (100X), and 1% 200 mM L-glutamine with media replacement every 48 h. Intestinal cells were plated in 12-well plates using a seeding density of 5 × 10^5^ cells/well and incubated at 37°C in a 5% CO_2_ humidified atmosphere for 21 days post-confluency. Media was replaced with serum- and antibiotic-free media 1 day prior to all experiments. All cell culture reagents were purchased from Thermo Fisher (Gibco).

### 2.5. Adhesion of *Bifidobacterium infantis* to HT29-MTX cells

Ten mL of diluted bacterial suspension (OD_600_ = 0.1) was transferred to a sterile tube, centrifuged (3,500 × g, 4°C, 10 min), and resuspended in an equivalent volume of serum- and antibiotic-free cell culture medium. HT29-MTX cells were incubated with 1.5 mL of this suspension for 3 h to mimic gastrointestinal transit time. Bacteria control samples without HT29-MTX cells were simultaneously incubated to account for changes in bacterial growth resulting from incubation in high glucose media. Intestinal cells were washed 4X with sterile PBS to remove non-adherent bacteria followed by addition of 1% Triton X-100 (v/v) at 4°C for 30 min to lyse intestinal cells and release adherent bacteria. Samples from each well were centrifuged (8,000 × g, 4°C, 10 min) and washed twice before resuspending in 1.5 mL of saline solution. Control bacteria and adhered bacteria were plated on MRSC agar plates to determine the proportion of adhered bacteria using the following equation: % adhesion = (CFU/mL of adhered bacteria/CFU of control bacteria) × 100%. Adhesion assays were carried out in 3 independent experiments, each containing 4 replicates per sample.

### 2.6. Measurement of ζ-potential

The ζ-potential, or surface charge, of the bacteria was measured as previously described by Ortega-Anaya et al. (24). Briefly, measurements of the bacterial cells (OD_600_ = 0.1) in sterile PBS were recorded using a NanoBrook 90 Plus instrument (Brookhaven Instruments, Holtsville, NY). The refractive indices (RI) for gram-positive *Bacillus thuringiensis* bacterial cells (1.528) and RI for water (1.33) were used (25). Ten measurements per sample from 3 independent experiments were collected at 25°C using the Smoluchowski approximation.

### 2.7. Transmission electron microscopy

The bacterial cells with or without MPL were prepared and washed as previously described. A concentrated suspension was prepared in PBS that was further mixed with sterile melted low-gelling temperature agarose solution (4% at 60°C). After solidification, small squares (1–2 mm^2^) were cut using a scalpel and were further fixed with 3% formaldehyde and incubated for 24 h. After carefully removing the supernatant, the samples were washed 5 times with PBS under gentle agitation (5 min each). Transmission electron microscopy (TEM) analysis was performed at the Campus Microscopy and Imaging Facility (CMIF) of The Ohio State University (OSU). Each sample was fixed with 2.5% glutaraldehyde for 2 h, post-fixed with 1% osmium tetroxide in 0.1 M phosphate buffer for 2 h, and then stained with 1% uranyl acetate for 1 h at room temperature. After dehydration in a graded ethanol series (50, 70, 80, 90, 100), they were embedded in Eponate 12 resin. The samples were sectioned at 70 nm using an ultramicrotome (EM UC7; Leica Microsystems, Vienna, Austria) and stained with 2% uranyl acetate and Reynold’s lead citrate. Then, they were visualized using a FEI Tecnai G2 Spirit microscope (FEI Company, United States) operated at 80 kV.

### 2.8. Growth and bound polysaccharide production curves

To determine the effect of MPL on bound polysaccharide production during bacterial growth, 20-μL of bacterial suspension in saline solution (OD_600_ = 0.1) was added to 180 μL MRSC with or without MPL in triplicate in a sterile 96-well plate. Each well was covered with 100 μL of sterile mineral oil for anaerobiosis. The OD_600_ was recorded every 2 h over 24 h in a 37°C microplate reader with 5 s of low-speed shaking prior to each read. Concurrently, 1 mL of the OD-adjusted bacterial suspensions were inoculated into 9 mL of MRSC with or without MPL under anaerobic conditions at 37°C for quantification of bound polysaccharides at 0, 4, 8, 12, 20, and 24 h. Separate tubes were prepared for each time point. At the end of incubation, the tubes were centrifuged (3,500 × g, 4°C, 10 min) and the pellets were washed with saline. Each pellet was subject to alkaline treatment using 2 mL of 2 M NaOH and agitated overnight at 25°C on a rocking platform to remove the bound polysaccharides while rendering the bacterial cells intact (26). The samples were centrifuged (7,000 × g, 4°C, 30 min) and the polysaccharides (supernatant) were precipitated with 2 volumes of EtOH with overnight incubation at −20°C. After centrifugation (9,000 × g, 4°C, 30 min), the crude polysaccharide pellets were resuspended in 0.5 mL MilliQ ultrapure water (18.2 MΩ • cm) and subjected to total sugar and protein quantification using the phenol sulfuric acid assay (27) and a Pierce micro-BCA kit (Thermo Fisher), respectively.

### 2.9. Functional group analysis using FTIR

Using MRSC agar plates with and without MPL, *B. infantis* was grown under anaerobic conditions for 48 h at 37°C using anaerobic gas packs. A loopful of bacteria was diluted and resuspended in 20 μL of EtOH. Then, 1 μL of this suspension was added to the diamond-ATR crystal of a 4500a Fourier Transform Infrared (FTIR) Spectrometer Portable Unit (Agilent Technologies, Inc., Santa Clara, CA) and dried using vacuum. This process was repeated several times until sufficient sample was applied to the instrument. The spectra of samples between 4,000 and 700 cm^−1^ were recorded using the MicroLab software (Agilent) using a resolution of 4 cm^−1^ and 128 scans. The spectra obtained were classified and analyzed using a SIMCA model with 2^nd^ derivative data deconvolution on Pirouette software V4.5 (Infometrix Inc., Woodville, WA).

### 2.10. Bound polysaccharide composition and quantification using HPLC-CAD

Bound polysaccharides were extracted from bacterial pellets of a 200 mL culture in the presence or absence of 0.5% MPL following the procedure of Ferrari et al. (26). Hydrolysis of extracted sugars was executed by resuspending the freeze-dried powder to a concentration of 12.5 mg/mL using sonication (10 min, 37°C). Resuspended sample was mixed with a half volume of 0.5 M H_2_SO_4_ and heated at 100°C for 40 min. The sample was then adjusted to pH 7 using 5 M NaOH and diluted to a final concentration of 2 mg/mL in reverse osmosis (RO) water. For quantification of monosaccharides by HPLC, 400 μL of sample was further diluted in 600 μL ACN. Separation of neutral monosaccharides was achieved using an XBridge Amide 3.5 μm (4.6 × 250 mm) column (Waters) equipped with a guard column of the same filling on a Dionex Ultimate 3000 HPLC system with a charged aerosol detector (CAD; Thermo Fisher) (28). Solvent A consisted of 50% IPA, 0.2% TEA, and 25 mM ammonium acetate in water and solvent B consisted of 90% ACN, 0.2% TEA, and 25 mM ammonium acetate. A flow rate of 0.5 mL/min, column temperature of 80°C, and isocratic elution of 90% B for 30 min were followed by column washing and re-equilibration. The CAD evaporator temperature was set to 35°C with a data collection rate of 10 Hz and filter constant of 1.0. All analytical standards and HPLC-grade solvents were purchased from Sigma. The final sugar quantification was normalized by CFU/mL.

### 2.11. Isolation of surface proteins

Surface proteins were extracted from a 30 mL culture of *B. infantis* in MRSC with or without MPL after washing in saline solution. Six mL of 5 M LiCl at 37°C for 60 min with agitation was used to remove non-covalently bound proteins (29, 30). Samples were centrifuged at 10,000 × g for 20 min at 10°C and the supernatant was subsequently dialyzed in Milli-Q water at 4°C to remove LiCl using cellulose dialysis tubing (MWCO 12 kDa, Sigma). Surface protein extracts were then concentrated using a Vacufuge Plus concentrator (Eppendorf, Enfield, CT) at 45°C and total protein was determined using a micro-BCA kit.

### 2.12. SDS-PAGE and LC-MS/MS

The surface protein profile of *B. infantis* in response to MPL was visualized using SDS-PAGE by loading 30 μg protein per sample on a Mini-Protean TGX Stain-free gel (4–20%) and applying 90 V for 15 min followed by 180 V for 30 min. Five μL of Precision Plus Unstained Protein Standard was used as a reference. Gels were imaged using a ChemiDoc MP Imaging System and densitometric analyses of the gels were performed using Image Lab Version 6.1 to semi-quantitatively determine differences in bacterial surface proteins. All reagents and equipment used for gel electrophoresis were purchased from Bio-Rad. Bands with the greatest increase or decrease in expression (at least 2-fold change) were excised, digested in gel using trypsin, and sequenced using Capillary-LC/MS/MS at the Campus Chemical Instrument Center (CCIC) Mass Spectrometry and Proteomics Facility at OSU. Data were searched using Mascot Daemon by Matrix Science V2.7.0 (Boston, MA) via Proteome Discoverer V2.4 (Thermo Scientific) and the database searched against the most recent UniProt databases. A decoy database was also searched to determine the false discovery rate (FDR) and peptides were filtered accordingly at 1% FDR. Proteins identified with at least two unique peptides were considered as a reliable identification. The mass spectrometry proteomics data have been deposited to the ProteomeXchange Consortium via the PRIDE (31) partner repository with the dataset identifier PXD042186. Although the proteomics was not performed in replicates, we did run 3 independent replicates of the SDS-PAGE gels and the band patterns exhibited were compared using the BioRad ImageLab software. The two bands of interest to us were present in the 3 independent replicates with similar experimental molecular weight.

### 2.13. Statistical analysis

All experiments were conducted at least in triplicate. Data presented are represented as the mean ± SEM where appropriate. Statistical analysis for ζ-potential, digestion survival, and monosaccharide composition were conducted using a Student’s *t*-test. Statistical analysis for adhesion was conducted using Mann Whitney *U*-tests. Differences of *p* < 0.05 were considered statistically significant. All statistical analysis was performed in GraphPad Prism V9.4 unless otherwise noted.

## 3. Results

### 3.1. MPL improve the survival of *Bifidobacterium infantis* during digestive stress and decrease adhesion to goblet-like cells

**Figure 1:**
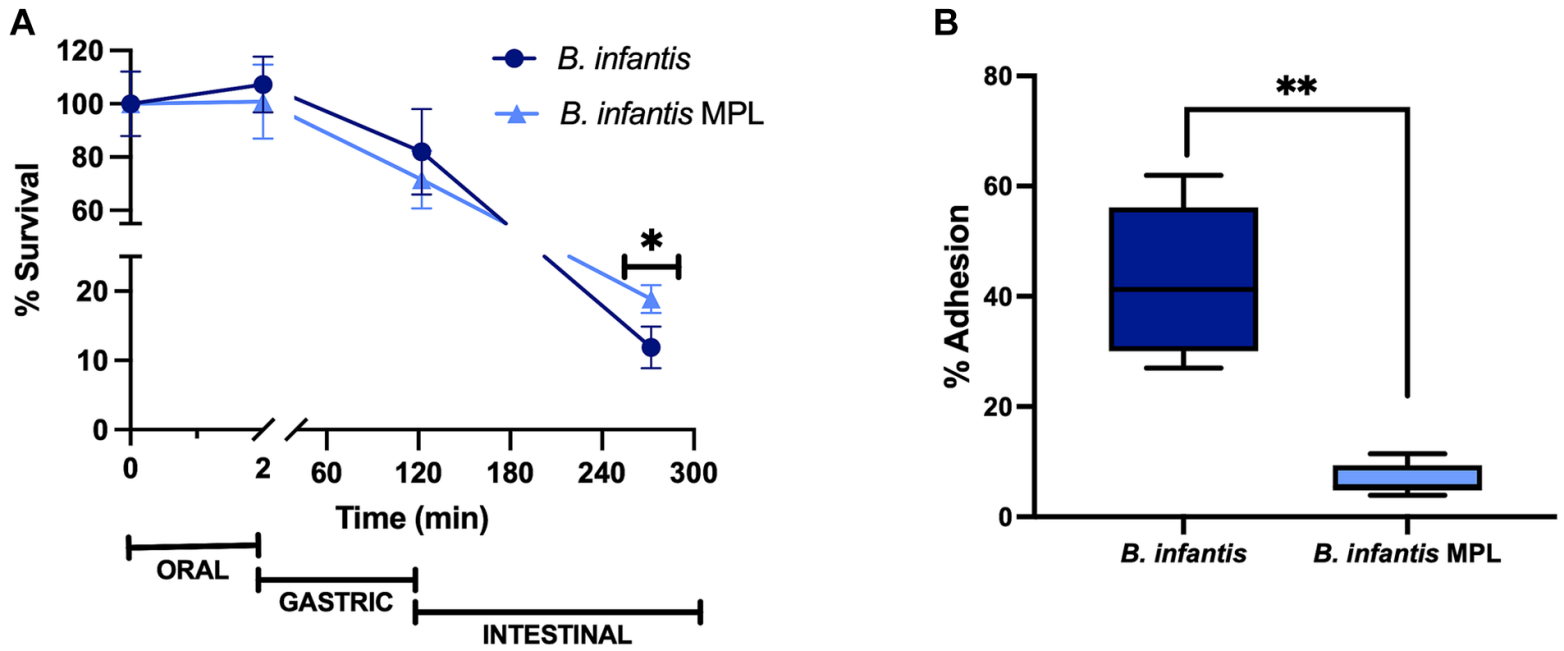
MPL prolongs the survival of *Bifidobacterium infantis* during simulated digestion and decreases adherence to mucus-secreting intestinal cells. **(A)** Survival of *B. infantis* with or without 0.5% MPL across the oral, gastric, and intestinal phases using the INFOGEST simulated digestion model. **(B)** Adhesion of *B. infantis* after incubation with 0.5% MPL to HT29-MTX cells. All experiments were performed at least in triplicate. Statistical difference between treatments is denoted by (^*^p < 0.05) or (^**^p < 0.0001) using a Student’s *t*-test and Mann–Whitney *U*-test, respectively.

Under simulated digestion conditions, MPL treatment significantly improved the survival of *B. infantis* at the end of the intestinal phase (*p* < 0.05) from 12% to 19% (Figure 1A). However, no changes in survival in the earlier digestive phases (oral and gastric) were observed in the presence of MPL. These findings suggest the role of MPL in mitigating bile-induced stress exhibited in the intestinal phase, while having no effect on low pH tolerance from gastric conditions.

As a follow up to these changes in survival, the influence of MPL bifidobacterial adhesion was tested using a cell culture model, HT29-MTX, which is a mucin-producing clone of HT29 cells and models goblet-like cells of the intestinal epithelium. This cell line permits the potential study of interactions between bacteria and mucus that are resemblant of the intestinal barrier. After MPL treatment, the bacteria were applied to the goblet-like cells for 3 h under aerobic conditions. The 3-h exposure resulted in a significant decrease in the adherence of *B. infantis* from 42.4 to 6.77%, when previously incubated with MPL (Figure 1B). This decrease corresponds to an approximately 0.2-log reduction. Using the same cell line, four inflammatory cytokines (IL-6, MCP-1, TNFα, and IL-4) were also measured in response to control or MPL-treated *B. infantis* and decreasing trends of expression were observed for pro-inflammatory mediators; however, no significant differences were detected (data not shown).

### 3.2. MPL decrease surface charge of *Bifidobacterium infantis* and modify surface morphology

**Figure 2:**
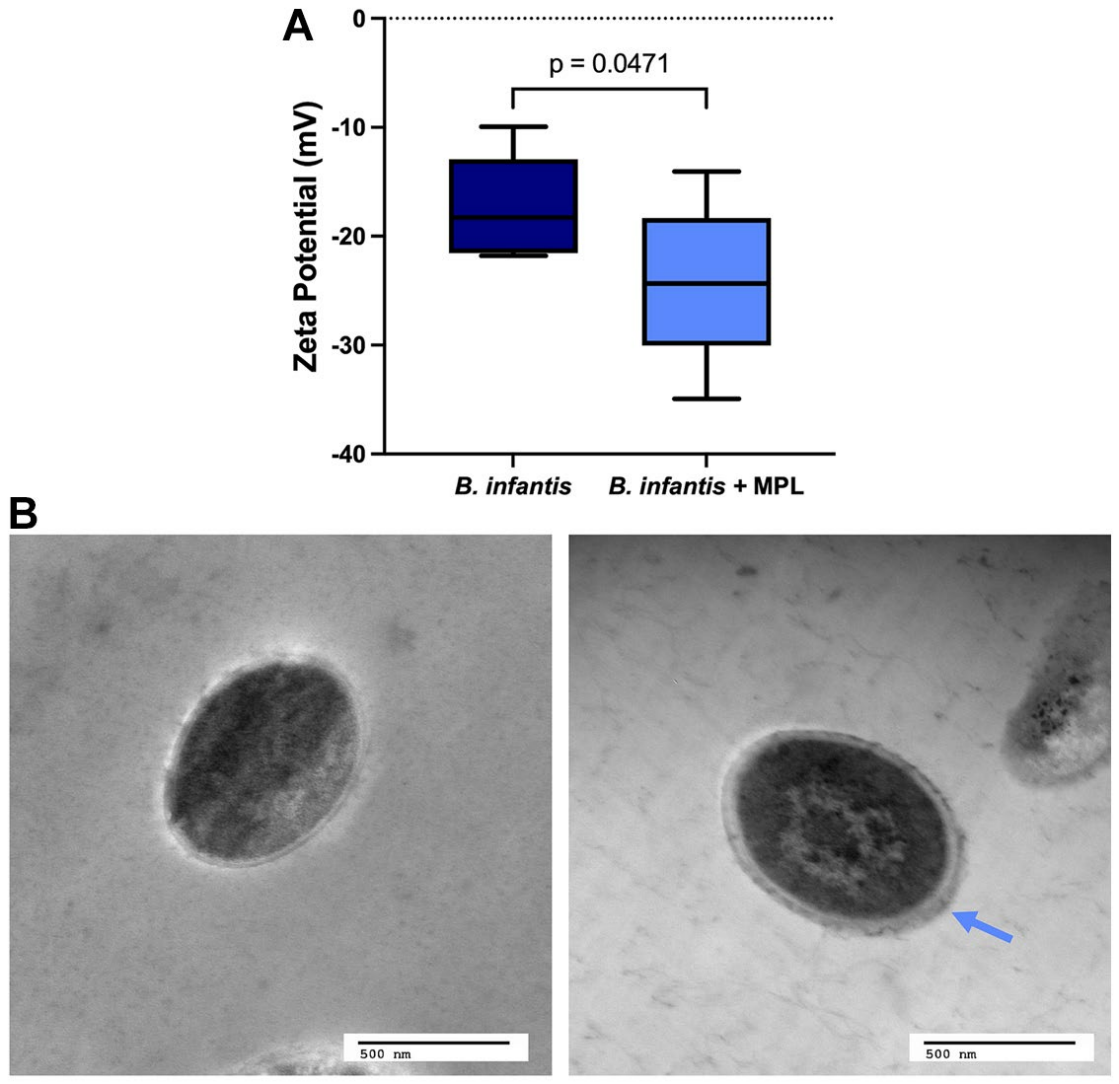
MPL induces surface changes on *Bifidobacterium infantis*. **(A)** ζ-potential, or surface charge, of *B. infantis* with or without 0.5% MPL. **(B)** Transmission electron micrographs of *B. infantis* (left) and *B. infantis* after MPL treatment (right). Blue arrow indicates thicker outer layer after MPL treatment. Statistical analysis was performed using a Student’s *t*-test (*p* < 0.05; *n* = 3).

The surface charge of gram-positive bacteria is generally attributed to the presence of cell surface proteins and carbohydrates, such as wall teichoic acid (WTA), lipoteichoic acid (LTA), and capsular polysaccharides, among others, that can be induced by changes in the environment and by nutritional stimuli (32). To estimate the surface charge of *B. infantis* in the presence and absence of MPL, ζ-potential was measured. The ζ-potential of *B. infantis* was found to be negative and decreased significantly upon exposure to MPL from −17.3 to −24.3 mV (Figure 2A). The decreased, or more negative, surface charge of *B. infantis* in response to MPL suggests attachment of MPL at the surface or an alteration in the composition of the cell surface, potentially through changes in surface carbohydrates and/or proteins.

TEM was used to investigate whether the observed changes in surface charge impacted the surface morphology of these bacteria. Figure 2 displays the TEM micrographs of *B. infantis* in the presence and absence of MPL. As indicated by the blue arrow in Figure 2B, the outer surface of *B. infantis* appears thicker upon MPL treatment, which resembles an increase in bacterial surface polysaccharides.

### 3.3. Increase in bound polysaccharides of *Bifidobacterium infantis* in presence of MPL

**Figure 3:**
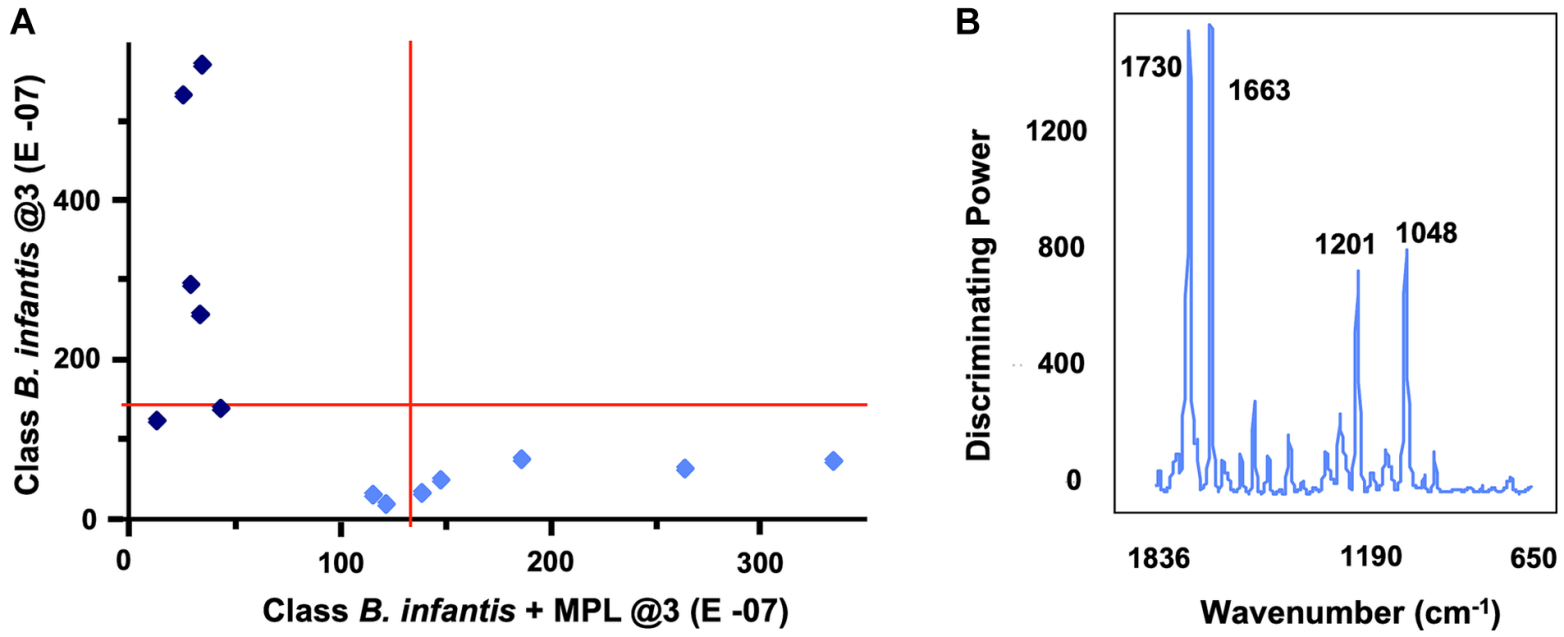
Cooman’s plot **(A)** depicting sample classification for “*Bifidobacterium infantis*” vs. “*B. infantis* + MPL” using soft independent modeling of class analogy (SIMCA) with three principal components. Vertical and horizontal lines show the 95% confidence intervals. *Bifidobacterium infantis* and *B. infantis* + MPL samples are represented by dark blue diamonds and light blue diamonds, respectively. Identification of SIMCA discriminating power **(B)** between MPL-treated and untreated *B. infantis* using the 2nd derivative of mid-IR spectra. Bacteria were suspended in ethanol and dehydrated using vacuum. Data acquisition was performed using a 4500a series FTIR spectrometer.

Considering the effect of MPL on the surface charge and morphology of *B. infantis*, an initial screening for features affected by the MPL treatment was conducted using FTIR. Bacterial colonies were resuspended in ethanol to reduce the interference of matrix components on the samples. The second derivative of the raw spectra was obtained to deconvolute overlapping bands while conserving the meaning and interpretation of specific wavenumbers. An assessment by soft independent modeling by class analogy (SIMCA) was used to identify the interclass distance and important bands with high discriminating power between the class “*B. infantis*” and the class “*B. infantis* + MPL.” Using three principal components, a Cooman’s plot was constructed to depict the orthogonal differences of each sample to the classification models (Figure 3A). The vertical red line indicates the cutoff point with samples being classified as “*B. infantis*” if to the left of the line. The horizontal red line indicates the cutoff point with samples being classified as “*B. infantis* + MPL” if below the line. Samples generally fit well within their respected classes, with a few exceptions belonging to either class (bottom, left quadrant; Figure 3A). Regardless, an interclass distance of 5.8841 was obtained, indicating the FTIR spectra of the classes were significantly different as values above 3 indicate statistical significance. To determine the most interesting bands generating these differences, the magnitude of the discriminating power for each band is represented in Figure 3B. The bands with the greatest discriminating power include 1,730, 1,663, 1,201, and 1,048 cm^−1^. The assignments and interpretations of these bands are listed in Table 1. The data collectively shows that MPL treatment is associated with changes in the lipid profile, suggesting the accumulation of MPL and changes in the bacterial surface through carbohydrate polymers.

**Table 1:** Assignments and identifications of discriminating FTIR bands between *Bifidobacterium infantis* control and *B. infantis* with MPL in the range of 4,000 cm^−1^ to 700 cm^−1^.

Band (cm^−1^)	Assignment	Component	Main corresponding cellular compounds	References
1,730	C=O stretching	Lipid esters	Membranes	(33–35)
		Lipid peroxides	Biofilm formation	
1,663	C=C stretching (Amide I)	Proteins	Membranes Cytoplasm	(33, 34)
			Surface proteins	
			Ribosomes	
1,201	C–O and C–C stretching; P=O stretching	Polysaccharides	Capsule	(33, 34)
		Phospholipids	Membranes	
		Phosphodiester bonds		
1,048	C–O–C stretching	Polysaccharides	Capsule	(33, 36)
			Exopolysaccharides	
			Peptidoglycan	

**Figure 4:**
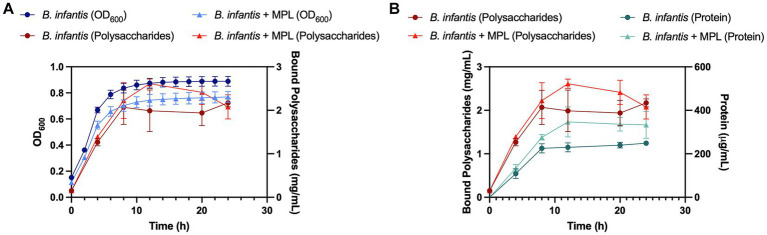
Growth (blue) and bound polysaccharide production (red) curves **(A)** for *Bifidobacterium infantis* with or without MPL treatment. Protein quantification (green) compared to bound polysaccharide production (red) curves **(B)** for *B. infantis* with or without MPL treatment. Experiments were conducted in triplicate.

We further explored the production of bound polysaccharides in the presence and absence of MPL by carbohydrate quantification across the growth of *B. infantis*. Figure 4A represents the quantification of bound polysaccharides over time through monitoring the *B. infantis* growth curve using OD_600_. Both control and MPL-treated bacteria exhibited similar exponential phase lengths (0 to 8 h); however, the carrying capacity was greater for control bacteria compared to the MPL-treated bacteria represented by the differences in OD_600_ in the stationary phase. During the exponential phase, no differences between control and MPL-bound polysaccharide production were observed. At the start of the early stationary phase (12 h), bound polysaccharide production peaked in both treatments but was higher in MPL-treated bacteria despite the overall lower cell count based on OD_600_ readings. This trend continues until 20 h, when production begins to decline likely due to the lack of remaining available nutrients, and eventually returns to similar levels as the control samples at 24 h.

Since carbohydrates from peptidoglycan and glycoproteins could possibly contribute to the observed increase in bound polysaccharides, the protein content was also monitored and compared to trends of bound polysaccharide production (Figure 4B). The data suggest that the observed increase in bound polysaccharides at 12 h is likely not related to the peptidoglycans and glycoproteins as the increase in protein did not mirror the increase in bound polysaccharides production. These findings were further corroborated by quantification of the neutral sugars galactose (D-Gal) and glucose (D-Glu), which are the principal sugars comprising *B. infantis* ATCC 15697 polysaccharides, using HPLC-CAD. Total Gal and Glu increased from 63.7 g/100 g powder to 110 g/100 g powder and retained similar D-Gal to D-Glu ratios near 1.6:1 with MPL treatment (Table 2). These data support the role of MPL in enhancing bound polysaccharides production in *B. infantis*.

**Table 2:** Monosaccharide composition of *Bifidobacterium infantis* bound polysaccharides using HPLC-CAD.

Treatment	Total sugars (Gal + Glu) g/100 g powder	D-Gal g/100 g powder	D-Glu g/100 g powder	Ratio Gal:Glu
*B. infantis*	63.7 ± 6.42^a^	38.4 ± 4.38^a^	25.3 ± 2.12^a^	1.5:1
*B. infantis* MPL	110 ± 8.67^b^	68.2 ± 4.61^b^	42.2 ± 4.47^b^	1.6:1

^*^Different letters within the same column indicate statistical significance (*p* < 0.05), Student’s *t*-test.

### 3.4. Differential *Bifidobacterium infantis* surface proteins in presence of MPL

**Figure 5:**
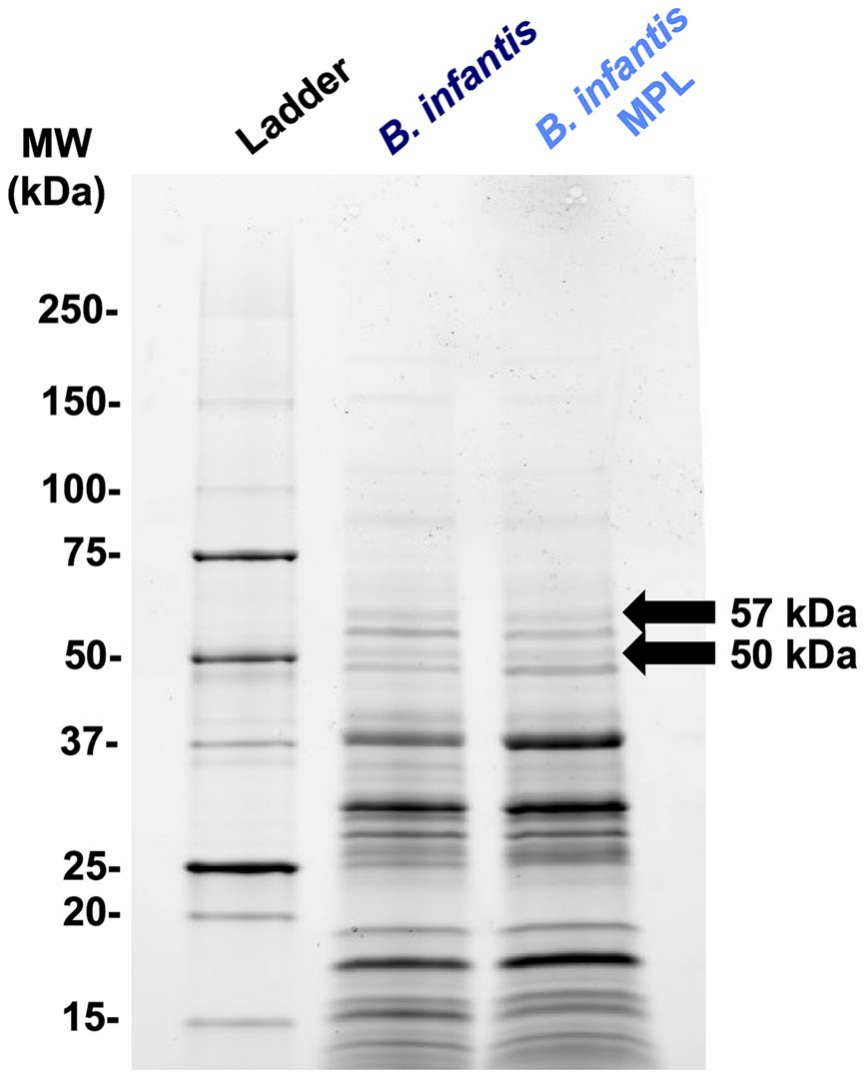
Representative SDS-PAGE (4%–20%) of *Bifidobacterium infantis* surface proteins extracted using 5 M LiCl with or without MPL. Arrows indicate differentially expressed protein bands at least 2-fold analyzed using densitometry.

For an additional level of understanding how MPL influences bifidobacteria surface properties, the effect of MPL on the surface protein profile was assessed as proteins also modulate bifidobacteria-host interactions within the GIT, and influence stress tolerance and GIT survival (37). Using SDS-PAGE separation (Figure 5), extracted surface proteins of control and MPL samples were compared using a densitometric assessment. Two bands with the greatest relative changes were excised and sequenced, as identified in Figure 5. These proteins were identified as a peptide ABC transporter substrate-binding protein with an experimental molecular weight (MW) of 57 kDa that decreased 2.02-fold in the presence of MPL and a trypsin-like peptidase domain-containing protein with an experimental molecular weight (MW) of 50 kDa that increased 3.36-fold (Table 3). Due to the nature of the SDS-PAGE separation, the discrepancy in experimental and theoretical MW of the trypsin-like peptidase domain-containing protein could be due reduction of disulfide bonds between subunits leading to a 50 kDa experimental MW compared to the theoretical 60 kDa MW. Furthermore, we noticed a tendency in the overlap between the sequenced peptide matches from this band and the accession protein toward the C-terminus.

**Table 3:** LC-MS/MS identification of *Bifidobacterium infantis* surface proteins in the presence or absence of MPL.

Relative band intensity								
*B. infantis*	*B. infantis* MPL	Fold change	Experimental MW (kDa)	Theoretical MW (kDa)	% coverage	LC-MS/MS identification	No. of peptides matched	Accession no.
3.50	1.73	−2.02	57	60	23%	Peptide ABC transporter substrate-binding protein	11	MBT8924194
0.46	1.53	3.36	50	60	14%	Trypsin-like peptidase domain-containing protein	2	WP_220433680

## 4. Discussion

Previous literature has shown that *B. animalis* has greater tolerance to acidic conditions than other *Bifidobacterium* strains (38). This phenomenon is reflected by only a 10%–20% decrease in survival of both control and MPL-treated bacteria at the end of the gastric phase in the present study. Resistance of this species to acidic conditions is attributed to an increase in the production of subunits of F0-F1-ATPases that counteract H^+^ accumulation and increased amino acids and ammonia production in the cytoplasm (39). Bile salt tolerance is dependent on the bile detoxification mechanisms like the efflux pumps betA and Ctr as bile salts are passively taken up by the bacteria (40). Additionally, the cell membrane is one of the main targets of bile salts and therefore the unique lipid, protein, and carbohydrate compositions of the bacterial cell wall can have a direct effect on bacteria bile salt tolerance. In this study, the 0.5% MPL treatment may have aided in the preadaptation of *B. infantis* to lipids similar to those present in bile and thus, contributed to their marginal increased survival at the end of the intestinal phase. Future experiments must expand these findings to higher concentrations of MPL to identify the dose–response behavior of these lipids, as well as determine whether higher concentrations offer greater protection.

Bacterial survival is also highly dependent on food matrices, which buffer and protect the bacteria due to the structural hindrance of the given food matrix. For example, the presence of sugars, non-bifidobacterial polysaccharides (i.e., alginates), and non-digestible carbohydrates enhance bacterial survival (11, 41). Recently, survival of 93% in a yogurt matrix was observed for *B. animalis* subsp. *lactis* BB-12 in the gastric phase, and 66% in the intestinal phase using the INFOGEST static digestion protocol (42). In the present study, we did not digest the bacteria within a food matrix to reduce sources of contamination and other interfering components in the experiment. However, we expect that within a food matrix, the bacterial survival will be even greater due to the protective effects of matrices.

The ability for bacteria to interact with the host is one significant mechanism by which beneficial bacteria convey their health benefits. Transient interactions with the host and adhesion to the intestinal epithelium occur through electrostatic and hydrophobic interactions between the bacteria and mucosa, specifically via bacterial proteins, like adhesins, and non-proteinaceous molecules, such as bacterial polysaccharides (39). The adhesion of *B. infantis* ATCC 15697 to HT29-MTX cells found in this study was similar to values reported for this species using other intestinal cell culture models (43, 44). As an alternative to cell culture-based adhesion measurements, estimating the surface charge through measurement of ζ-potential has been proposed as a useful method for predicting adhesion between bacteria and the intestinal mucosa, as there appears to be a positive relationship between surface charge and adhesion (24). Changes in bacterial surface electronegativity can also translate to lipid-mediated cell signaling, membrane permeability, and metabolite uptake within the bacteria (45, 46). In this study, the surface charge of *B. infantis* was found to be negative and similar to other reported values in the literature for bifidobacteria and other gram-positive bacteria (47, 48). Both surface charge and adhesion decreased upon MPL treatment, which may be related to the increase in bifidobacterial polysaccharides that are negatively-charged in nature (49). This relationship is reinforced by previous studies in which an increase in *Lactobacillus johnsonii* surface charge was correlated to increases in adhesion to chicken gut explants (50). These findings were explained by a reduction in EPS production. Aside from reduced adhesion, the reduced EPS layer was also associated with decreased survival during GIT transit, corresponding well to the changes observed in the present study. Using CPS knockout mutants of *B. longum* 105-A, Tahoun and colleagues (51) identified these surface polysaccharides as a critical factor in bifidobacterial survival against GIT stressors, including tolerance to acidic pH and resistance to bile salts, as well as a key modulator of adhesion to intestinal cells. Although the precise mechanisms of bacterial polysaccharides modulating adhesion have yet to be fully elucidated, EPS seems to promote probiotic persistence rather than colonization in the intestinal tract by allowing them to reach the colon alive and evade the host’s innate immune defenses (52, 53). Specifically, increased EPS may have a role in shielding the bacterial adhesins to evade B cell responses, which further prolongs their persistence in the colon (54).

The effect of milk components on probiotic adherence to intestinal cells appears to be multifactorial in terms of bacterial species, strain, bacteria culture medium, growth phase, dairy ingredient, and its composition, among others (24, 44, 47, 55–57). In the present study, *B. infantis* treated with MPL adhered to intestinal cells, albeit at a lower proportion than control samples. The same phospholipid ingredient, PL700, used in this study has been shown to increase adhesion in *Lacticaseibacillus paracasei, Pediococcus acidilactici,* and *Limosilactobacillus reuteri* in Caco-2 cells, but had no effect on other strains (55). Using a co-culture model of Caco-2 and HT29 cells, the same ingredient increased surface electronegativity was correlated to increased adhesion of lactic acid bacteria to intestinal cells in a strain-dependent manner (24). MFGM-rich butter serum significantly decreased the adhesion of *Lacticaseibacillus rhamnosus* GG by preventing the interactions between SpaCBA pili and Caco-2 TC7 intestinal epithelial cells. The authors emphasize that the milk protein fraction of the MFGM may be responsible for inhibiting this interaction (58). However, milk proteins were not detected in the PL700 ingredient (data not shown), suggesting an alternative explanation for adherence interference, such as adsorption of phospholipids on the bacteria cell surface or changes in surface protein expression. However, we cannot rule out that the observed decreased in adhesion could be a combination of changes in surface protein expression and polysaccharide expression as well as adsorption of phospholipids on the surface of the bacteria.

In the present study, we identified changes in two surface proteins (Figure 5), a substrate-binding protein and a peptidase domain-containing protein. Substrate-binding proteins work as the functional surface domains of ABC transporters in prokaryotes and are widely present in Gram positive bacteria. Depending on the class of the ABC transporter, they can transport various ligands (i.e., amino acids, peptides, carbohydrates) driven by H^+^ or Na^+^ gradients (59). The peptidase domain-containing protein identified by 3.4-fold change contains a degP htrA domain, which is reportedly involved in the protection of bacteria from thermal and other types of stress (60). The ortholog in *Pseudomonas aeruginosa* is found in an operon that controls the mucoid phenotype. The potential increase in expression of this protein may provide a rationale for the increase in survival during simulated digestion after MPL treatment.

It has been reported that *B. longum* ssp. *infantis* ATCC 15697 produces two types of capsular polysaccharides—one unbranched and with repeating units of D-Gal and D-Glu in a 3:1 ratio and one with a main chain of galactose with 90% of galactofuranose and 30% galactopyranose subunits (5). In the present study, we quantified neutral sugars and observed a D-Gal to D-Glu ratios of 1.6:1 (Table 2). This is not surprising since the culture conditions and medium composition greatly influence carbohydrates yield, composition, and ratio (5). The polysaccharides of bifidobacteria have been associated with the direct modulation of the gut microbiota communities. Inside the gut, bifidobacterial polysaccharides are thought to act as prebiotic substrates for fermentation by resident gut microbes (61). Using *B. breve* UC2003 and its EPS knockout, Püngel and colleagues (62) demonstrated the role of EPS in influencing the abundances of microbial populations associated with infant microbiotas (i.e., *Tyzzerella* and *Faecalibacterium*) and their metabolism through short chain fatty acid production. Others have shown that two *B. animalis* subsp. *lactis* strains with differing EPS chain length and compositions have distinctive impacts on gut microbiota composition as well as circulating cytokine levels in murine models (63). Bifidobacterial polysaccharides are recognized by the TLR2 and TLR4 receptors of the host, blocking these recognition sites for pathogens, and activating signaling pathways that could lead to production of anti-inflammatory cytokines (64). However, it is still unclear whether the binding affinity is dependent on molecular weight or structure of the polysaccharides. Due to the attributed health-associated properties of *Bifidobacterium* polysaccharides and their role in the development and maintenance of the gut microbiota, the findings that MPL increase polysaccharides production appears to be beneficial to the host.

An interesting follow-up to this study would be the determination of which specific MPL classes have the most significant protective effects on *B. infantis* relating to enhanced survival and polysaccharide production. Many dairy waste streams, including buttermilk from butter processing and whey from various cheeses, are unique sources of MPL that mimic the ratios and proportions of phospholipid classes found in the MFGM. Utilization of waste streams such as these is economically favorable and accessible from an industrial perspective; however, the identification of specific PL classes involved in the bifidobacterial protective effects would aid the elucidation of their mechanism(s) of action on the bacterial cell.

## 5. Conclusion

Bifidobacteria are appealing supporters of human health through their wide range of functions in the GIT. In line with the recent interest in utilizing dairy components to enhance the probiotic and postbiotic traits of beneficial bacteria, we investigated the effect of MPL on survival of *B. longum* subsp. *infantis* ATCC 15697 during digestive stress and its ability to adhere to intestinal cells. Prolonged survival during simulated digestion combined with the increase in bacterial bound polysaccharides and surface protein changes suggest that MPL may be capable of promoting desirable functional properties through bacterial EPS production in foods and/or the digestive tract. This highlights the possibility for novel enhancement of *B. infantis* probiotic and postbiotic potential using dairy ingredients. The current applications of bifidobacterial polysaccharides as constituents in foods are limited due to their low yield at the end of production, but the potential applications of MFGM-related ingredients as inducers of bacterial polysaccharides may offer new solutions to these challenges. This study substantiates future bifidobacteria-containing product development that supports human health and wellness.

## Data availability statement

The data presented in the study are deposited in the PRIDE repository, accession number PXD042186.

## Ethics statement

Ethical approval was not required for the studies on humans in accordance with the local legislation and institutional requirements because only commercially available established cell lines were used. Ethical approval was not required for the studies on animals in accordance with the local legislation and institutional requirements because only commercially available established cell lines were used.

## Author contributions

EK, BG-O, IG-C, JO-A, and RJ-F contributed to conception and design of the study. EK, BG-O, and JO-A performed the experiments and conducted the analyses. EK and BG-O drafted the manuscript. All authors contributed to the article and approved the submitted version.
