# Supplementary material for: Milk phospholipids protect Bifidobacterium longum subsp. infantis during in vitro digestion and enhance polysaccharide production
Source: Front Nutr. 2023 Nov 6;10:1194945. doi: 10.3389/fnut.2023.1194945 (PMC10657999; doi:10.3389/fnut.2023.1194945)
Supplement: Supplementary file 1 [file Table_1.DOCX]

Supplementary Material

Milk phospholipids protect *Bifidobacterium longum* subsp. *infantis* during *in vitro* digestion and enhance polysaccharide production

Jiménez-Flores, R.*, Kosmerl, E., González-Orozco, B., García-Cano, I., Ortega-Anaya, J.

* Correspondence:

Rafael Jiménez-Flores
jimenez-flores.1@osu.edu

# Supplementary Table

| **Table S1.** Phospholipid and fatty acid composition of the PL700 ingredient. | | |
| --- | --- | --- |
| Phospholipid Classes (mg per 1 g of powder) | | |
|  | PC | 158.2 |
|  | PE | 114.0 |
|  | PI | 27.9 |
|  | PS | 18.5 |
|  | SM | 174.9 |
| Fatty Acid Composition (% of Total Lipids) | | |
|  | C4:0 | 0.243 |
|  | C6:0 | 0.167 |
|  | C8:0 | 0.085 |
|  | C10:0 | 0.278 |
|  | C12:0 | 0.887 |
|  | C14:0 | 5.02 |
|  | C15:0 | 0.831 |
|  | C16:0 | 20.3 |
|  | C16:1 | 1.38 |
|  | C17:0 | 0.464 |
|  | C18:0 | 13.2 |
|  | C18:1 Trans-9 | 3.14 |
|  | C18:1 Cis-9 | 42.1 |
|  | C18:2 Trans-9,12 | 0.583 |
|  | C18:2 Cis-9,12 | 5.52 |
|  | C20:1 Cis-11 | 2.09 |
|  | C18:3 | 2.629 |
|  | C22:0 | 0.330 |
|  | C22:1 Cis-13 | 0.657 |
